# Association of folate metabolism-related genetic polymorphisms with susceptibility to breast cancer: A protocol for a systematic review and meta-analysis

**DOI:** 10.1097/MD.0000000000026926

**Published:** 2021-08-13

**Authors:** Chuan Wang, Chuan-Ming Tong, Yuan Zhang, Guo-Xiu Chen, Feng Xiong, Ju Wang

**Affiliations:** Department of General Surgery, People's Hospital of Dongxihu District, Wuhan, Hubei, China.

**Keywords:** breast cancer, folate metabolism, genetic polymorphisms, meta-analysis

## Abstract

**Background:**

Breast cancer has recently become one of the most common causes of cancer-related deaths, and several studies have suggested that genetic polymorphisms in the folate metabolism pathway may be associated with susceptibility to breast cancer, although their results have been inconsistent or inconclusive. Therefore, the aim of this meta-analysis was to obtain accurate, consistent conclusions regarding the potential associations of genetic polymorphisms in the folate metabolism pathway with the risk of breast cancer, based on case-controlled studies.

**Methods:**

From the beginning of database establishment through May 2021, we indexed and searched domestic and foreign databases, including the Chinese National Knowledge Infrastructure, Web of Science, VIP and BioMedical Database of China, PubMed, EMBASE, Wanfang database, and the Cochrane Library. To determine the effects of folate metabolism-related genetic polymorphisms on breast cancer risk, we used Stata version 16.0 to analyze all data and calculated variable odds ratios and 95% confidence intervals.

**Results:**

The findings of the current meta-analysis are going to be presented to peer-reviewed journals for publication when the analysis is completed.

**Conclusion:**

The meta-analysis will summarize the association of genetic polymorphisms in the folate metabolism pathway with breast cancer.

**Registration number:**

May 26, 2021.osf.io/25r48. (https://osf.io/25r48/).

## 1 Introduction

Breast cancer is one of the most common malignancies among women; based on the GLOBOCAN estimate, there were approximately 2.3 million newly diagnosed cases of breast cancer and 0.69 million associated deaths globally in 2020.^[1]^ The underlying biology of cancer development and progression is complex, and the causes of breast cancer are not entirely clear, although hormone replacement therapy, obesity, and radiation have been identified as factors associated with the risk of breast cancer.^[2]^ However, not all people exposed to these carcinogenic factors will eventually develop cancer, suggesting that genetic factors may also play key roles in cancer development. Folic acid and vitamin B12 are 2 key elements in single-carbon metabolic pathways, and genetic polymorphisms in the folate metabolism pathway may play important roles in cancer development.

The relationships between genetic polymorphisms in the folate metabolism pathway and the risk of breast cancer have attracted much attention in recent years, and have been evaluated by many studies.^[3,4]^ Although many molecular and epidemiological studies have explored the associations of folate metabolism related genetic polymorphisms with the risk of breast cancer, no consensus has been reached.^[5]^ Moreover, because of genetic polymorphisms in the folate metabolism pathway, as well as nationality-related, regional, and racial differences, the results from different studies may vary, and even contradictory findings may be reported. Accordingly, such data should be comprehensively evaluated using a meta-analysis to verify the association between genetic polymorphisms in the folate metabolism pathway and breast cancer susceptibility.

## 2 Methods

### 2.1 Study registration

The review protocol was registered with OSF (registration number: DOI 10.17605/OSF.IO/25R48) following the guidelines for the declaration of preferred reporting projects for meta-analysis protocols and systematic reviews.

### 2.2 Inclusion criteria

The inclusion criteria were as follows: studies assessing the relationships between folate metabolism-related genetic polymorphisms and breast cancer were analyzed and evaluated; the sample scale, 95% confidence interval (CI), and odds ratio (OR) were provided; and for multiple publications from the same research group, the most complete and recent results were adopted.

### 2.3 Exclusion criteria

The exclusion criteria were as follows: articles published with incomplete or incorrect information, and incomplete correspondence information provided; articles reporting data obtained from nonhuman subjects; and literature without related outcome indicators.

### 2.4 Search strategy

We selected the key words “breast cancer,” “genetic polymorphisms,” and “folate metabolism” in Chinese as search terms to check Chinese databases, including the Wanfang Database, VIP and BioMedical Database of China, and Chinese National Knowledge Infrastructure. The key words “folate metabolism,” “polymorphisms,” “breast tumor,” and “breast cancer” were used when searching English databases, including the Cochrane Library, Web of Science, PubMed, and EMBASE. Literature from domestic and international databases were obtained from the date of inception through May 2021.

### 2.5 Collection and analysis of data

#### 2.5.1 Study selection

**Figure 1:**
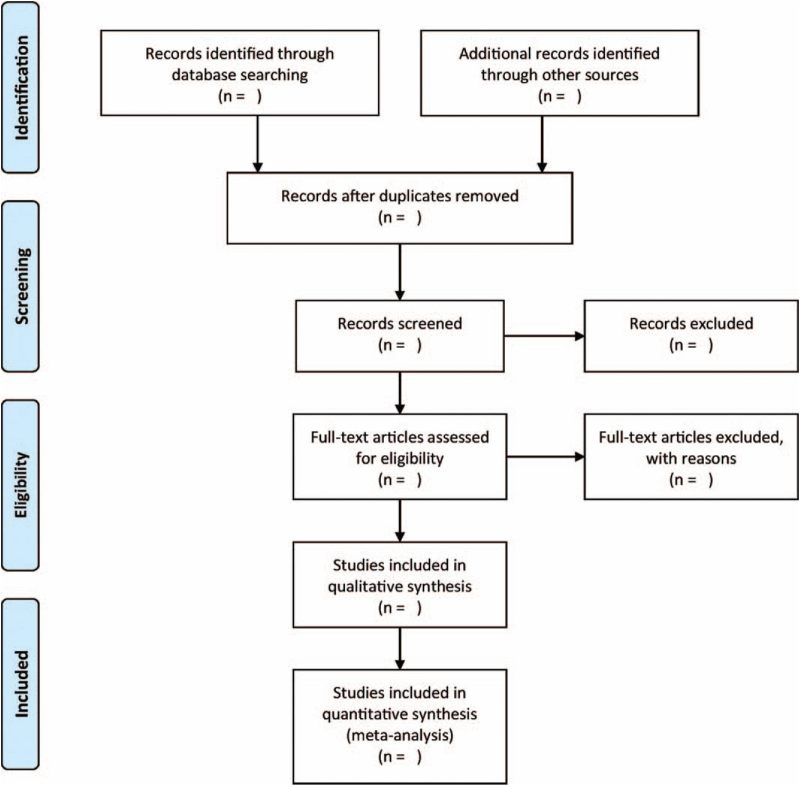
Flowchart for the meta-analysis.

The process of study selection, which involved finding titles, reading abstracts, and scanning full texts to determine whether the articles met the inclusion criteria, was conducted independently by 2 reviewers. Any discrepancies were reconciled by a third reviewer. We excluded the conclusions from each study according to the preferred report item records in the systematic review and guidelines for meta-analyses. The flow chart of the study selection process is displayed in Figure 1.

#### 2.5.2 Data extraction

Two independent researchers selected all the data sources that were included in the analysis, and discrepancies were resolved through discussion. The following information was gathered from the studies: first author, country of study, publication year, characteristics of patients and controls (e.g., race, age), control and case group types, genotyping method, genotype frequency, and Hardy--Weinberg equilibrium *P* value for the control group.

#### 2.5.3 Methodology quality assessment

The quality of the included studies was assessed independently by two reviewers based on the Newcastle--Ottawa Scale, which evaluates the quality of observational studies by scoring them on a scale of 0 to 9 points. The quality of the studies was considered reliable if the total score was more than 6 points.

#### 2.5.4 Subgroup analysis

We performed a subgroup analysis of the relationships of genetic polymorphisms in the folate metabolism pathway with breast cancer susceptibility according to ethnicity and genotyping.

#### 2.5.5 Sensitivity analysis

We used Stata version 16.0 for sensitivity analysis, and the stability of the meta-analysis results was verified after using another exclusion method.

#### 2.5.6 Assessment of publication bias

Publication bias was analyzed using Egger linear regression and Begg rank correlation. A *P* value of less than .05 indicated a significant publication bias.^[6,7]^

### 2.6 Ethical considerations

Ethical approval was not required because this was a meta-analysis.

## 3 Discussion

As its incidence has increased worldwide, breast cancer has become one of the most common diseases among women; however, the causative factors have not been fully elucidated.^[8]^ Previous studies have suggested that folate has important roles in regulating DNA repair and is involved in the modulation of DNA methylation.^[9,10]^ Moreover, genetic polymorphisms in folate-metabolizing enzymes, such as methionine synthase (MTR), MTR reductase, and 5,10-methylene tetrahydrofolate reductase, can lead changes in folic acid levels.^[11–13]^ Therefore, genetic polymorphisms in these folate-metabolizing enzymes may also affect DNA repair, or lead to abnormal DNA methylation, resulting in genetic instability and promoting the development of breast cancer. The meta-analysis conducted herein was performed to assess the relationships between folate metabolism-related genetic polymorphisms and breast cancer susceptibility; our findings will provide insights into the diagnosis and treatment of breast cancer.

## Author contributions

**Conceptualization:** Chuan Wang, Guo-Xiu Chen.

**Data curation:** Chuan Wang, Yuan Zhang, Guo-Xiu Chen, Feng Xiong.

**Formal analysis:** Chuan Wang, Chuan-Ming Tong, Feng Xiong.

**Funding acquisition:** Chuan Wang, Yuan Zhang, Ju Wang.

**Investigation:** Chuan-Ming Tong, Feng Xiong, Ju Wang.

**Methodology:** Chuan Wang, Chuan-Ming Tong, Yuan Zhang, Guo-Xiu Chen.

**Project administration:** Ju Wang.

**Resources:** Chuan-Ming Tong, Yuan Zhang, Guo-Xiu Chen.

**Software:** Chuan Wang, Chuan-Ming Tong, Feng Xiong.

**Supervision:** Chuan Wang, Chuan-Ming Tong.

**Validation:** Chuan Wang, Feng Xiong, Ju Wang.

**Visualization:** Yuan Zhang, Guo-Xiu Chen, Feng Xiong.

**Writing – original draft:** Chuan Wang, Ju Wang.

**Writing – review & editing:** Ju Wang.
